# **Bioimpedance** analysis combined with sagittal abdominal diameter for abdominal subcutaneous fat measurement

**DOI:** 10.3389/fnut.2022.952929

**Published:** 2022-08-10

**Authors:** Chung-Liang Lai, Hsueh-Kuan Lu, Ai-Chun Huang, Lee-Ping Chu, Hsiang-Yuan Chuang, Kuen-Chang Hsieh

**Affiliations:** ^1^Ministry of Health and Welfare, Department of Physical Medicine and Rehabilitation, Puzi Hospital, Chiayi, Taiwan; ^2^Department of Occupational Therapy, Asia University, Taichung, Taiwan; ^3^General Education Center, National Taiwan University of Sport, Taichung, Taiwan; ^4^Department of Oral Hygiene, Tzu-Hui Institute of Technology, Pingtung, Taiwan; ^5^Department of Orthopedics, China Medical University Hospital, Taichung, Taiwan; ^6^Ministry of Health and Welfare, Department of Physical Medicine and Rehabilitation, Taichung Hospital, Taichung, Taiwan; ^7^Department of Research and Development, Starbia Meditek Co., Ltd., Taichung, Taiwan; ^8^Big Data Center, National Chung-Hsing University, Taichung, Taiwan

**Keywords:** abdominal obesity, bioelectrical impedance, cross-validation, sagittal abdominal diameter (SAD), anthropometric

## Abstract

Abdominal subcutaneous fat tissue (ASFT) is an independent predictor of mortality. This prospective observational study aimed to establish a rapid, safe, and convenient estimation equation for abdominal subcutaneous fat area (SFA) using bioimpedance analysis (BIA) combined with sagittal abdominal diameter (SAD). A total of 520 adult subjects were recruited and were randomly divided into 2/3 (*n* = 346) and 1/3 (*n* = 174) to form a modeling group (MG) and a validation group (VG), respectively. Each subject's abdomen was scanned using computed tomography to obtain target variables (SFA_CT_). Predictor variables for all subjects included bioimpedance index (h^2^/Z), anthropometric parameters height (h), weight (W), waist circumference (WC), hip circumference (HC), and SAD, along with age and sex (male =1, female = 0). SFA estimation equation SFA_BIA+SAD_ was established for the MG using stepwise multiple regression analysis. Cross-validation was performed using VG to evaluate the performance of the SFA_BIA+SAD_ estimation equation. Stepwise multiple regression analysis was applied from the MG, including SFA_BIA+SAD_ = 49.89 + 1.09 SAD−29.90 Sex + 4.71 W−3.63 h^2^/Z−1.50 h (*r* = 0.92, *SEE* = 28.10 cm^2^, *n* = 346, *p* < 0.001). Mean differences in SFA_BIA+SAD_ relative to SFA_CT_ were −1.21 ± 21.53, 2.85 ± 27.16, and −0.98 ± 36.6 cm^2^ at different levels of obesity (eutrophic, overweight, obese), respectively. This study did not have a large number of samples in different fields, so it did not have completely external validity. Application of BIA combined with SAD in anthropometric parameters achieves fast, accurate and convenient SAF measurement. Results of this study provide a simple, reliable, and practical measurement that can be widely used in epidemiological studies and in measuring individual SFA.

## Introduction

Obesity has become a global medical problem over the past few decades, and is projected to only worsen in the foreseeable future (1). Overweight and obesity are closely associated with chronic disease and increased morbidity and mortality. Related issues often lead to cardiovascular disease or metabolic syndrome. Direct and indirect medical expenses also place a huge economic burden on society (2).

Abdominal subcutaneous fat tissue (ASFT) and abdominal visceral adipose tissue (AVAT) have different effects on metabolic homeostasis, but their quantity and distribution are both risk factors for cardiometabolic diseases (3). Abdominal obesity is a major risk factor for diabetes and cardiovascular disease. Excess visceral and subcutaneous fat are key contributors to abdominal obesity. Visceral and subcutaneous fat differ in structure, metabolic activity, and functional significance. The current study suggests that a positive caloric balance in individuals with impaired adipogenesis may lead to adipocyte hypertrophy of the ASFT. This in turn leads to impaired energy storage and ASFT dysfunction (4). Insufficient ASFT reservoirs can lead to redistribution of free fatty acids to ectopic tissues such as liver, and skeletal muscle, thereby increasing metabolic risk (5). Metabolic syndrome may develop when ASFT stores fat in ectopic locations, which may lead to developing insulin resistance or lipotoxicity (6, 7). When AVAT is higher than ASFT, the risk of atherosclerotic cardiovascular disease and hemodynamic abnormalities increases (8, 9).

Several methods for indirect assessment of abdominal fat include body mass index (BMI), waist-to-hip ratio (WHR), and skin-fold thickness measurement (10–12) or dual-energy X-ray absorptiometry (DXA) (13). However, to obtain more accurate measurements, non-invasive methods such as computed tomography (CT) or magnetic resonance imaging (MRI) are required, which can accurately identify specific fat areas. Several studies have compared CT and MRI techniques (14, 15), and the use of MRI or CT to measure abdominal fat has been validated since the 1980 s (16, 17). Recent fully automated CT and MRI can accurately measure visceral and subcutaneous fat in obese people (18, 19). However, the high cost and time requirements of CT and MRI are the main limitations of their widespread use even though they yield precise measurements when performing medical examinations or academic research.

Bioelectrical impedance analysis (BIA) is a simple, safe, rapid, and non-invasive method for assessing body composition. Many studies have compared the measurement results of the calibration method and BIA, and they are widely used in clinical and epidemiological studies (20, 21). BIA can also be used to measure AVAT or visceral fat area (VFA) (22, 23) but the application of BIA to the measurement of ASFT or abdominal subcutaneous fat area (SFA) is very limited. Therefore, this study applied bioimpedance measurement combined with anthropometry, and used computed tomography as a reference method to establish and verify the estimation equation of BIA in SFA.

## Subjects and methods

### Study design and subjects

In this prospective observational and cross-sectional study. Subjects were recruited through hospital advertisements and word of mouth at Puzi Hospital in southern Taiwan. The subjects were tested by the non-random purposive sampling method. Potential participants were healthy adults who came to the hospital for their free NHS continuing healthcare checklist (24). Answering questionnaires and signing experimental consent forms was under the introduction of a trained research assistant. Long-term bedridden persons, those who had a change in weight in the previous year or recently, and those who had undergone abdominal surgery in the past were excluded from this experiment. Patients with malignant tumors and chronic liver disease were also excluded from this experiment. A total of 520 subjects were ultimately included in the study. This study complied with the ethical guidelines of the 1975 Declaration of Helsinki. Participants filled out personal data, including medical history and health status. Included subjects were adults over age 20 years who were free from endocrine, nutritional or growth disorders, or any major chronic diseases. Subjects previously diagnosed with diabetes, cancer, liver disease with renal insufficiency, or chronic asthma or pregnancy were excluded. The 520 included subjects were randomly divided into groups of 2/3 (*n* = 346) and 1/3 (*n* = 174) to form a MG and a VG, respectively.

### Ethical considerations

The study protocol was approved by the Human Trials Committee of Taso-Tun Psychiatric Center, Ministry of Health and Welfare, Nan-Tou, Taiwan (IRB 109043). After volunteers met the inclusion criteria, and received explanation of the study from the researchers, all included subjects provided signed informed consent to participate.

### Anthropometry

Participants' body weights were measured to the nearest 0.1 kg using a body composition analyzer BC418MA (Tanita Co, Tokyo, Japan). Each subject's barefoot height was measured to the nearest 0.5 cm using a height ruler (Holtain, Cosswell, Wales, UK). Body mass index (BMI) was defined as weight (kg) divided by height (meters) squared. Waist Circumference (WC) is measured with the feet together, the abdominal muscles relaxed, the arms naturally on the sides, normal breathing, and the narrowest position of the body below the ribs and above the navel. Hip Circumference (HC) is measured at the widest point if the buttocks (25); both were measured using a standard tape measure to the nearest 0.1 cm. Each anthropometric measurement was performed by trained observers. All subjects wore hospital cotton/polyester blend gowns and minimal underwear. The measuring tool was calibrated weekly by the same observer.

### Computerized tomography

Participants' abdominal region was scanned using a 64-slice computed tomography scanner (Somatron Sensation 64 CT System, Siemens Corp., Germany) together with operating software (software version, Syngo CT2005A). Each participant was placed supine on the CT scanning platform, the lumbar region was scanned, with scanning voltage 120 Kv, tube current 120 mAs, X-ray width 1.5 mm, scanning time 0.5 s, slice thickness 5 mm, and images were captured at 2 mm intervals. The image reconstruction kernel index was B20.

**Figure 1:**
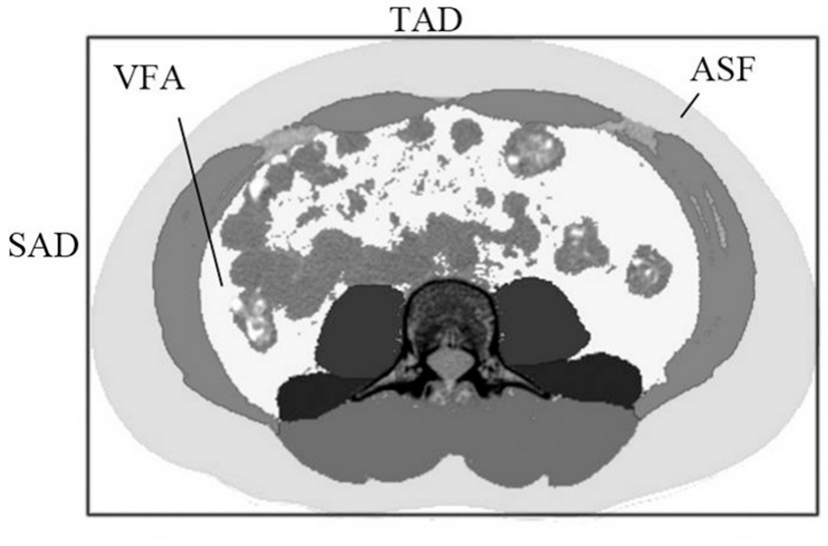
The sagittal abdominal diameter (SAD) and the transverse abdominal diameter (TAD) in the abdominal cross-sectional image, with subcutaneous adipose tissue and visceral adipose tissue represented in light gray and white, respectively.

Two image analysts (observers) were trained by radiologists in localizing human anatomy in relation to the L3–L4 lumbar spine. A fixed image analysis program was used to calculate the abdominal cross-sectional area (ACSA) at the L3–L4 lumbar height. Image processing Slice-O-Matic version 4.3 software (Tomovision, Magog, QC, Canada), and Slice-O-Matic image analysis program was used for quantitative analysis of the image area. The image format was DICOM (Digital Imaging and Communications in Medicine) and Slice-O-Matic was used for image opening. Image analysts used Slice-O-Matic to circle the VFA and SFA, expressed in VFA_CT_ and SFA_CT_. Sagittal abdominal diameter (SAD) was measured at the largest supine anteroposterior dimeter at lumbar vertebra levels of L3–L4. Transvers abdominal diameter (TAD) is defined as the largest spanned width of the body in the sliced image. As shown in Figure 1, its diameter was measured by manually fitting the smallest possible rectangle, including the entire abdominal area in the image slice.

### Bioelectrical impedance

Subjects were not allowed to drink alcoholic beverages 48 h before the test. No diuretics were used for the seven days prior to the test. Subjects had to fast for 4 h and avoid vigorous activity and alcohol for 24 h before the test. On arrival, subjects were asked to remove all metal objects and empty their bladder. After standing up for at least 10 min, subjects were placed on the BIA with barefoot and arms separated from the trunk. Thumbs, hands, feet and heels were positioned in contact with the corresponding electrodes. Two measurements were taken from each individual. For accurate measurements, wet hands and feet with an electrolyte paper towel before measurement. Female subjects were excluded from testing during menstruation. All subjects had no history of nutritional, endocrine or growth disorders. A standing 8-contact electrode impedance analyzer BC418MA (Tanita Co., Tokyo, Japan) was used for impedance measurements. During the test, the subject stands on the base platform, holds the handles embedded with the transmitting and sensing electrode plates with both hands, and the soles of the feet naturally touch the sensing and transmitting and sensing electrode pairs with body weight pressure. The BC418MA applies a single alternate current of 0.55 mA with the frequency of 50 kHz to measure the impedance of the left and right upper limbs, lower limbs and whole body, respectively.

The coefficient of variation of the impedance measurements of the current flow path throughout the whole body was evaluated within-day and between-days. Five males and five females were tested. The subjects repeated the impedance measurement 10 times within 1 h of the day, and the impedance measurement was carried out at the same time period over 5 days.

### Statistical analysis

Values in this study are presented as mean ± SD. Values shown in parentheses are the minimum and maximum values. Continuous variables in this study include weight, height, age and BMI and all of them were normally distributed according to the Shapiro-Wilk tests. Levene's test was performed to test its homogeneity. The Akaike Information Criterion (AIC) were presented to evaluate the precision of the estimation equations. Given that a sample size of 218 subjects was calculated considering a power of 95% and a type 1 error of 5% to achieve a medium effect size for the coefficient of determination (*r*^2^) increases in the estimation equation with the inclusion of 5 predictors (G^*^power 3.1) (26), or sample size of 346 athletes was sufficient for assuring an adequate power analysis in model development. Multiple linear regression analysis with stepwise variable selection, and SFA_CT_ was used as the response variable in the MG. The bioimpedance index “h^2^/Z” was combined with the anthropometric parameters of height (h), weight (W), age (Age), gender (sex, female = 0, male = 1), WC, HC, BMI, WHR, SAD and TAD were predictor variables. The parameters Forward (F_in_ = 4.00) and Backward (F_out_ = 3.99) were used to obtain the selected predictor variables. When the correlation between predictor variables was too high, variance inflation factor (VIF) ≥5 was applied to remove the predictor variables from the estimation equation. The estimation equation SFA (SFA_BIA+SAD_) was constructed, and the corresponding regression coefficient, standard error of the estimate (*SEE*), and *r*^2^ were obtained to evaluate the performance of the estimation equation. The SFA_BIA−SAD_ obtained by applying VG data was analyzed by correlation and Bland-Altman plot with SFA_CT_ as the reference value. BMI was the obesity criterion. All subjects were divided into three groups, eutrophic (BMI <25 kg/m^2^), overweight (25 kg/m^2^ ≤ BMI < 30 kg/m^2^), and obese (BMI > 30 kg/m^2^). One-way ANOVA was used to compare the differences of SFA_BIA+SAD_ and SFA_CT_ between the different obesity groups. All statistical analyses were performed using the statistical analysis software SPSS Version 20 (IBM SPSS, Armonk, NY, USA). The level of statistically significant difference was set at *p* < 0.05.

## Results

Subjects were randomly divided into an MG of 346 subjects and a VG of 174 subjects. The MG included 206 males (age: 37.8 ± 15.9 years old, BMI: 26.1 ± 3.5 kg/m^2^), 140 females (age: 41.3 ± 16.6 years, BMI: 24.9 ± 3.8 kg/m^2^). The VG included 107 males (age: 37.1 ± 14.9 years, BMI: 26.0 ± 3.6 kg/m^2^) and 67 females (age: 42.4 ± 16.8 years, BMI: 25.1 ± 3.6 kg/m^2^). The abdominal subcutaneous fat area (SFA_CT_) was 100.1 ± 68.3 cm^2^ in males and 142.0 ± 73.4 cm^2^ in females. The continuous variables were normally distributed (weight, height, age, and BMI). The measurement results for SFA_CT_ and other variables are shown in Table 1. For whole-body impedance measurements within 1 day, the coefficient of variation of the subjects was 0.3–0.8%. The coefficient of variation for the same subjects on the between-days was 0.9–1.8%.

**Table 1:** Demographic and physical characteristics of the study participants.

**Modeling group (*****n*** = **346)**					
**Variable**	**Males (*****n*** = **206)**		**Females (*****n*** = **140)**		***P***
Age (year)	37.8 ± 15.9	(18.2, 81.5)	41.3 ± 16.6	(18.5, 73.5)	^**^
Height (cm)	171.7 ± 7.2	(151.2, 197.3)	160.7 ± 6.0	(149.0, 176.0)	^**^
Weight (kg)	77.1 ± 12.1	(50.5, 124.5)	63.7 ± 1.1	(45.5, 110.2)	^**^
BMI (kg/m^2^)	26.1 ± 3.5	(19.7, 41.4)	24.9 ± 3.8	(18.8, 39.5)	^*^
ACSA_CT_ (cm)	488.0 ± 123.7	(286.3, 854.3)	432.7 ± 98.2	(273.4, 702.5)	^**^
VFA_CT_ (cm)	63.4 ± 48.5	(15.2, 201.4)	49.6 ± 33.4	(16.3, 192.5)	^**^
SFA_CT_ (cm)	99.0 ± 69.6	(12.2, 443.3)	145.2 ± 76.5	(45.7, 488.3)	^**^
WC (cm)	82.9 ± 10.1	(65.0, 122.0)	79.7 ± 1.6	(58.0, 122.0)	^*^
HC (cm)	98.4 ± 7.5	(65.0, 123.0)	97.3 ± 8.8	(61.5, 129.0)	
WHR	0.84 ± 0.07	(0.72, 1.24)	0.82 ± 0.07	(0.65, 1.09)	
Z (ohm)	523.1 ± 61.0	(372.0, 729.0)	663.7 ± 79.7	(506.0, 856.5)	^**^
H^2^/Z (cm^2^/ohm)	57.3 ± 8.2	(38.7, 81.8)	39.5 ± 5.2	(30.1, 51.8)	^**^
SAD (cm)	19.2 ± 2.6	(14.8, 29.8)	18.5 ± 2.8	(14.1, 30.1)	^*^
TAD (cm)	29.8 ± 3.7	(25.6, 37.7)	29.6 ± 3.9	(22.5, 40.6)	
**Validation group (*****n*** = **174)**					
	**Male (*****n*** = **107)**		**Female (*****n*** = **67)**		***P***
Age (years)	37.1 ± 14.9	(20.0, 61.1)	42.4 ± 16.8	(20.3, 84.8)	^**^
Height (cm)	172.6 ± 8.0	(148.0, 195.0)	159.9 ± 6.7	(143.3, 176.0)	^**^
Weight (kg)	77.8 ± 13.6	(43.2, 106.7)	63.8 ± 11.0	(43.2, 109.5)	^**^
BMI (kg/m^2^)	26.0 ± 3.6	(19.2, 37.9)	25.1 ± 3.6	(18.2, 37.9)	^*^
ACSA_CT_ (cm^2^)	483.0 ± 109.7	(294.3, 820.5)	432.1 ± 95.3	(280.4, 6,952)	^**^
VFA_CT_ (cm^2^)	65.2 ± 46.6	(17.2, 194.4)	49.6 ± 31.7	(17.3, 182.1)	^**^
SFA_CT_ (cm^2^)	102.1 ± 66.1	(12.0, 349.3)	135.4 ± 66.4	(30.8, 342.9)	^**^
WC (cm)	82.7 ± 10.3	(64.0, 124.0)	79.1 ± 1.8	(56.0, 120.0)	^*^
HC (cm)	98.8 ± 7.9	(62.0, 125.0)	96.3 ± 8.3	(62.1, 128.8)	
WHR	0.83 ± 0.06	(0.71, 1.22)	0.81 ± 0.06	(0.64, 1.10)	
Z (ohm)	528.2 ± 59.6	(411.3, 554.7)	668.1 ± 83.7	(496.6, 892.3)	^**^
h^2^/Z (cm^2^/ohm)	57.3 ± 8.3	(32.5, 66.0)	39.0 ± 6.3	(27.2, 58.6)	^**^
SAD (cm)	19.2 ± 2.7	(13.8, 26.5)	18.3 ± 2.8	(13.9, 24.9)	^*^
TAD (cm)	29.7 ± 3.1	(25.3, 36.7)	29.7 ± 3.7	(23.7, 39.3)	

*WHR, Waist-hip ratio; SAD, Sagittal abdominal diameter; ACSA, abdominal cross-sectional area; BMI, Body mass index; SFA, subcutaneous fat area; VFA, visceral fat area; h, height; TAD, Transvers abdominal diameter;*

* *p < 0.05;*

** *p < 0.001*.

In multiple linear regression analysis with stepwise variable selection, TAD, WC, HC, age, and BMI were excluded, and SAD, Sex, W, h^2^/Z, and h were predictor variables of SFA_CT_ in sequence. When each estimated variable was added to the SFA_CT_ estimation equation one by one, its *r*^2^, *SEE* and the regression coefficient of each estimated variable changed, as shown in Table 2. The SFA_BIA+SAD_ estimation equation is shown in equation (1):


(1) $$\begin{array}{l} \;{\text{SFA}}_{\text{BIA+SAD}}\text{ = }2.08\text{ SAD }-57.26\text{ Sex + }1.39\text{ W}\\ \;-3.67h^{\text{2}}\text{/}Z\;-\;1.50\;h\\ \left(r^{\text{2}}\text{ = }0.842,SEE\text{ = }28.10cm^{\text{2}},\;p\;<\;0.001,\;n\text{ = 346}\right) \end{array}$$

**Table 2:** Multiple regression analysis of sagittal abdominal diameter (SAD) measured with bioelectrical impedance measures as predictor variable and SFA_CT_ as response variable (Modeling group).

**Cumulative dependent variable used in model (*****n*** = **346)**								
**SAD**	+ **Sex**	+ **W**	+ **h**^2^**/Z**	+ **h**	**Intercept**	**SEE**	*r* ^2^	**ACI**
2.08 ± 0.09 (1.00)^**^	–	–	–	–	−277.17 ± 17.10^**^	44.12	0.612	854
2.26 ± 0.07 (1.03)^**^	−57.26 ± 3.86 (1.03)^**^	–	–	–	−276.83 ± 12.64^**^	34.40	0.764	832
1.82 ± 0.09 (2.17)^**^	−72.31 ± 4.38 (1.47)^**^	1.39 ± 0.22 (2.82)^**^	–	–	−277.82 ± 12.65^**^	32.62	0.788	812
1.39 ± 0.9 (2.75)^**^	−36.69 ± 5.44 (2.86)^**^	3.82 ± 0.32 (4.60)^**^	−3.67 ± 0.39 (4.70)^**^	–	−199.48 ± 14.02^**^	29.08	0.831	801
1.09 ± 0.11 (3.88)^**^	−29.90 ± 5.43 (3.06)^**^	4.71 ± 0.36 (4.81)^**^	−3.63 ± 0.37 (4.71)^**^	−1.50 ± 0.30 (3.11)^**^	43.89 ± 50.69	28.10	0.842	798

*Regression coefficient estimate ± SEE (VIF, variance inflation factor); r^2^, determination coefficient; SAD, sagittal abdominal diameter; SEE, standard estimate error; h^2^/Z, bioimpedance index; h, height;*

* *, P < 0.05;*

** *, P < 0.001; r^2^, coefficient of determinations; Sex (female = 0, male = 1); W, weight; AIC, Akaike's information criteria*.

**Figure 2:**
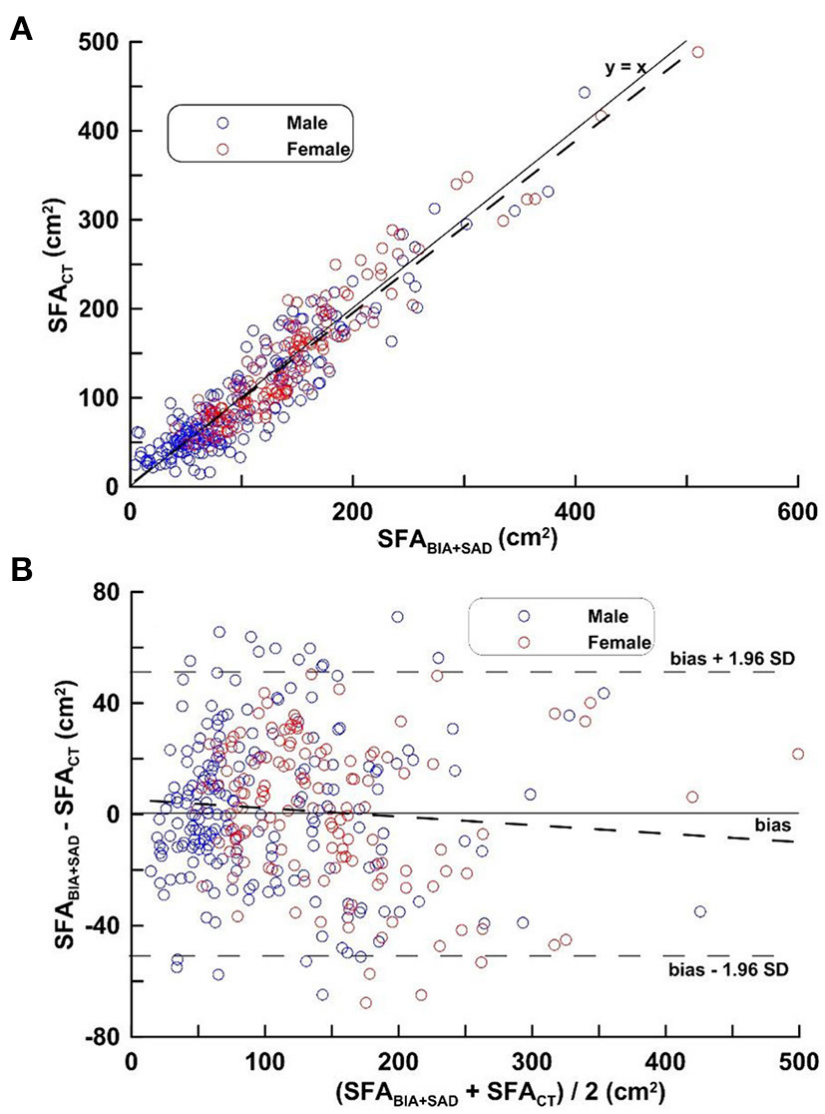
**(A,B)** Correlation analysis (top) and difference analysis (bottom) of SFA in the MG on SFA_CT_, bioimpedance and SAD. The difference (calculated SFA_CT_-SFA_BIA+SAD_ on Bland-Altman) is the mean of the corresponding measurements of SFA on CT and BIA + SAD (*y* = 12.07 − 0.029 x, *p* = 0.103). Blue circles represent males and red circles represent females.

Figure 2 depicts the regression line obtained by equation (1), the distribution and its mean difference, the limit of agreement (LOA) in the distribution diagram and the Bland-Altman Plots in the MG. In Figure 2A, the regression line equation is SFA_CT_ = 1.014 SFA_BIA+SAD_−2.341; in Figure 2B, Bias ± 1.96 SD was −51.29 and 51.32 cm^2^.

**Figure 3:**
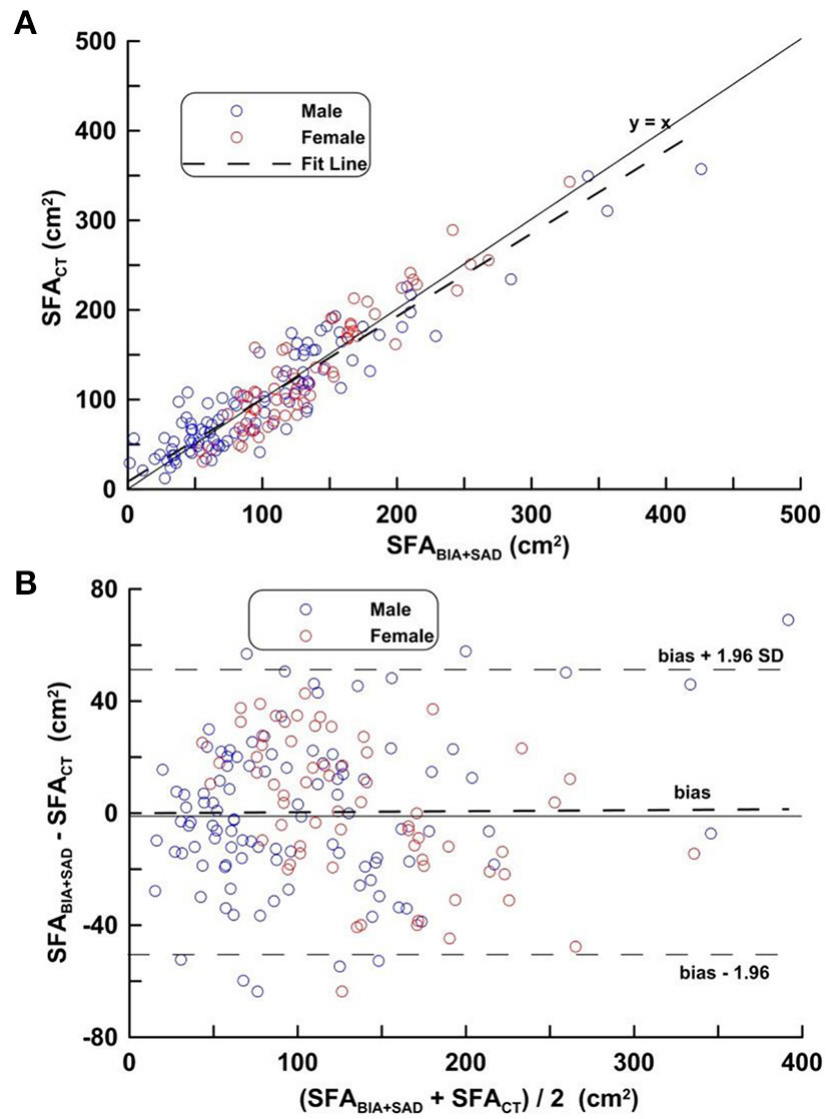
**(A,B)** Correlation analysis (top) and difference analysis (bottom) of SFA in the validation group on SFA_CT_, bioimpedance and SAD. The difference (calculated SFA_CT_-SFA_BIA+SAD_ on Bland-Altman) is the mean of the corresponding measurements of SFA on CT and BIA + SAD (*y* = 13.33 − 0.035, *p* = 0.193). Blue circles represent males and red circles represent females.

Figure 3 shows the regression line obtained by applying equation (1), the distribution and its mean difference, the LOA on the distribution plot and the Bland-Altman Plots on the VG. In Figure 3A, the regression line equation is SFA_CT_ = 0.972 SFA_BIA+SAD_−5.72, *r* = 0.930. In Figure 3B, bias ± 1.96 SD was −50.23, 50.67 cm^2^.

**Figure 4:**
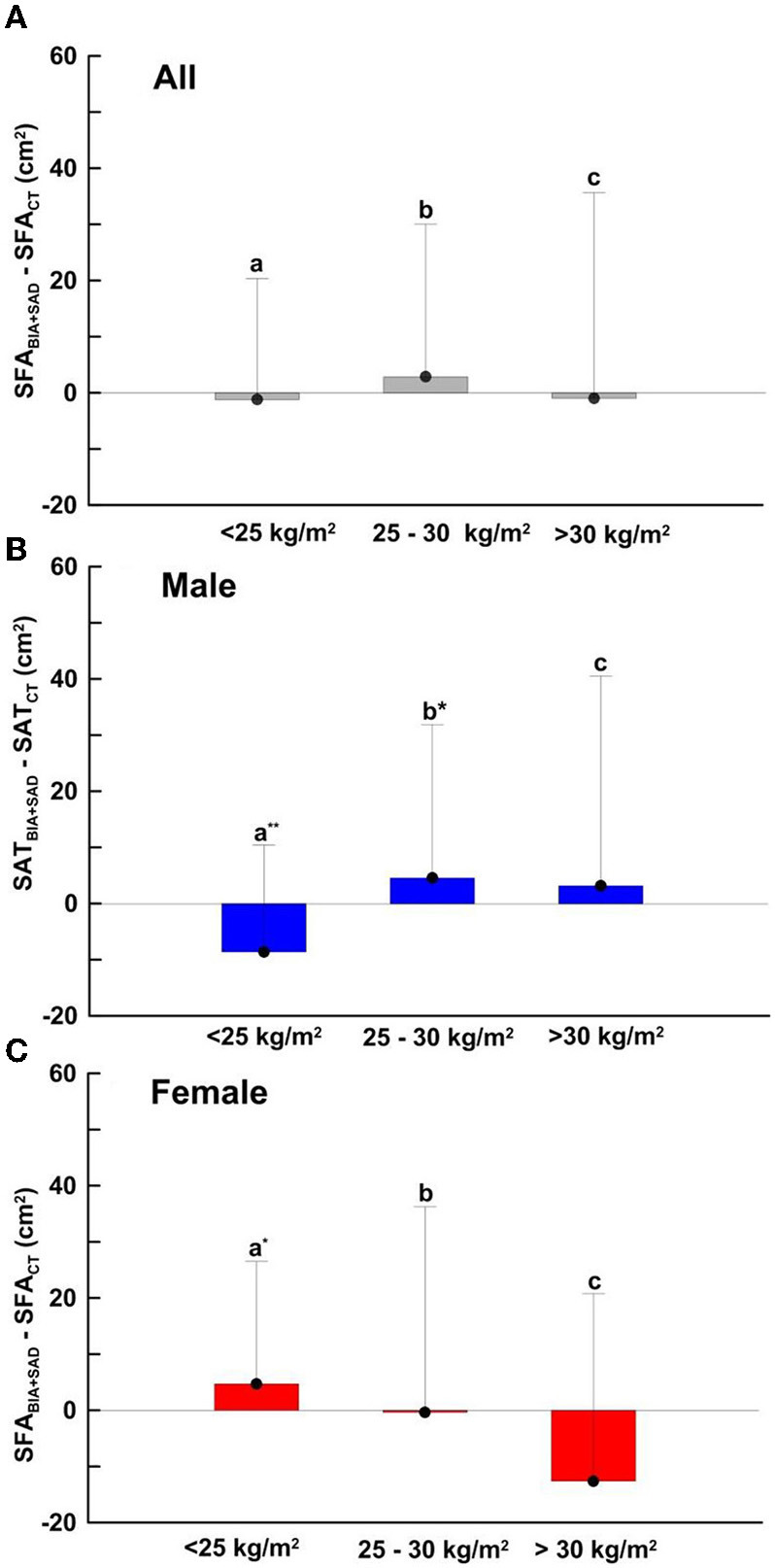
SFA-dependent bias of SFA_BIA+SAD_ compared with SFA_CT_ in **(A)** total (*n* = 520), BMI < 25 kg/m^2^, *n* =154; 25 kg/m^2^ ≤ BMI > 30 kg/m^2^, *n* = 275; BMI > 30 kg/m^2^, *n* = 104 **(B)** male (*n* = 310), BMI < 25 kg/m^2^, *n* = 67; 25 kg/m^2^ ≤ BMI > 30 kg/m^2^, *n* = 180; BMI > 30 kg/m^2^, *n* = 62 and **(C)** female (*n* = 210), BMI < 25 kg/m^2^, *n* = 86; 25 kg/m^2^ ≤ BMI > 30 kg/m^2^, *n* = 96; BMI > 30 kg/m^2^, *n* = 22. Data are presented as the mean difference ± SD. Means with symbol are significantly different, *p* < 0.05(*), *p* < 0.01(**).

Figure 4, Bar charts of Equation (1) was fitted from the three groups of eutrophic nutritional status (BMI < 25 kg/m^2^), overweight (25 kg/m^2^ ≤ BMI < 30 kg/m^2^), and obese (BMI > 30 kg/m^2^). As shown in Figure 4A, the mean differences of SFA_BIA+SAD_ and SFA_CT_ in the three groups were −1.21, 2.85, −0.98 cm^2^ and SD were 21.53, 27.16, 36.60 cm^2^, respectively. As shown in Figure 4B, the mean differences in the male group (Males, *n* = 309) were −8.62, 4.54, and 3.15 cm^2^, respectively, and the SDs were 19.01, 27.32, and 37.37 cm^2^, respectively. As shown in Figure 4C, the mean differences in the female group (Females, *n* = 204) were 4.70, −0.36, −12.61 cm^2^, and the SDs were 21.8, 36.6, 33.36 cm^2^, respectively.

The correlation coefficients between the response variable SAF_CT_ and each estimated variable were SAD (*r* = 0.782), WC (*r* = 0.750), BMI (*r* = 0.738), HC (*r* = 0.690), W (*r* = 0.516), h^2^/Z (*r* = −0.321), WHR (*r* = 0.287), sex (*r* = −0.265), h (*r* = −0.262), TAD (*r* = 0.220), and age (*r* = 0.216).

## Discussion

The present study is the first to use BIA combined with accurate abdominal computed tomography anthropometric parameters to verify the established SFA estimation equation with homogeneity. The current estimates of abdominal fat or abdominal obesity are based primarily on AVAT. Compared with AVAT, the current methods for estimating SFA are limited (27). Therefore, it is particularly important to establish a simple, safe and valuable SFA estimation equation for abdominal obesity or SFA measurement.

Many studies have shown that SFA correlated highly with anthropometric indicators such as WC, HC and BMI. However, results have been inconsistent across studies, ranging from 0.23 to 0.92 for the correlation of SFA with anthropometric measures (28, 29). This discrepancy may be due to the different characteristics of subjects in different age groups, ethnic groups or in different studies. In the stepwise regression analysis used in this study, SAD, gender, weight, bioimpedance index, and height were obtained sequentially as the predictor variables of SFA_CT_. However, age, WC, HC, BMI, and TDA were not selected as predictor variables in the SFA estimation equation in the stepwise regression analysis. SAD was the estimated variable that correlated most with the response variable “SFA” in this study, followed by WC, BMI, and HC. In fact, SAD was the first variable to be selected in stepwise regression analysis, which has the highest correlation with SFA_CT_ and is also the most explanatory of SFA among all predictor variables. WC, BMI, and HC had high collinearity with SAD, and the correlation coefficients were 0.825, 0.821, 0.668, respectively, but were not selected in the estimation model. This study reported a correlation coefficient of 0.78 between SAD and SFA, regardless of gender, which is similar to previous studies showing that the correlation coefficients between SAD and SFA were 0.66–0.78 and 0.72–0.76 in male and female, respectively (30–32). SAD acts as an indicator for estimating abdominal obesity and is highly correlated with cardio metabolic risk factors, anthropometric parameters and body fat estimates (33).

The bioimpedance index has good power and correlation with the body's fat-free mass or lean mass or body fluids (34). In the present study, the power or correlation of the bioimpedance index to SFA_CT_ was lower than that of many anthropometric variables. The power of the bioimpedance index for total body fat mass was also not high. The impedance measurement used in this study was a standing whole-body measurement mode. The dual-impedance method (35), which directly measures abdominal impedance, should theoretically increase the correlation with SFA, and may improve the measurement accuracy of BIA in SFA. VFA estimate has been provided by some of the BIA models using abdominal dual BIA or segmental BIA methods. The Inbody 720/770 model measures impedance of five segments of the body and VFA estimate is derived from a regression analysis using the segmental impedance. In contrast, a DUALSCAN HDS-2000 (Omron Healthcare Co., Kyoto, Japan), an abdominal dual BIA, provides a direct measurement of VFA. For dual abdominal BIA, electric current is applied to the limb electrodes for calculating fat-free mass and to the eight abdominal surface electrodes for calculating subcutaneous fat thickness with subject in a supine position (36). Compared to the dual abdominal BIA method, this study provided a more convenient way to measure SFA with subjects in a standing position. Furthermore, this study showed that SAD was highly correlated with SFA and can be used to predict VFA.

Potential variables for model fitting were age, sex, W, BMI, WC, HC, TAD, SAD and h^2^/Z in this study. TAD, WC, HC, age and BMI were excluded from the model during variable selection process. Finally, variables selected for the best regression model for estimating SFA_BIA_ included SAD, Sex, W, h^2^/Z. Age, sex, W, BMI, WC, HC, TAD and SAD have been shown to be correlated with SFA by previous studies. This study further identified h^2^/Z as a negatively correlated variable of the SFA_BIA_ estimation equation. The existing research literature rarely explored the relationship between SFA and h^2^/Z, except for the DUALSCAN HDS- 2000 study (35). The above findings are also another contribution of this study.

In the SFA estimation model of the present study, the estimated variable that best reflected SFA was SAD, while the traditional measurement of SAD was measured with calipers (32). However, with the substantial increase in computing power, artificial intelligence and image processing power, 3-dimensional optical body scanner (3DO) could be used in this study to replace CT in future applications to obtain accurate anthropometric parameters. 3DO provides a fast, widely integrated method for automated body composition estimation (37, 38). Therefore, in the future, the standing BIA measurement model combined with the standing 3D image body scanner could be used to integrate the predictor variables such as bioimpedance index and anthropometric measurements. We would expect this quick, automatic method to be widely used in SFA estimation.

In the present study, the standing position was used to measure the impedance of the whole body. Compared with the traditional supine impedance measurement, the impedance value obtained for the same measurement path or part was significantly different. The gravity factor of the standing impedance measurement mode affects the distribution of water in the human body, which in turn affects the impedance value of the measurement site. An average difference of about 10 ohms is found in the standing body or right hand to foot impedance values relative to the supine impedance. Standing impedance measurements decrease with time as the standing measurement time increases (39). Compared with traditional supine impedance measurement, standing bioimpedance measurement still has its limitations.

Among the existing anthropometric methods for SFA, the established measurement mode may have a good correlation coefficient, but the number of people who established the model was either too small or cross-validation was not performed (11). Therefore, the application value of related measurement methods is limited. In this study, 520 subjects were used in the MG (346) and the VG (174), and cross-validation was performed. Statistical indicators such as correlation, SEE, and LOA in MG or VG all showed that the SFA estimation model established in this study had a certain reference value. In addition; this study specifically explored the estimation error in different genders and different obesity levels, possibly making the results of this study more valuable than those from similar studies.

In this study, we used random sampling instead of stratified sampling to divided subjects into two groups. Since our study sample was homogeneous, our random sampling yielded homogeneous samples. In addition, studies used the same tools and methods and were controlled. We also run a Levene's test to test whether two groups have equal variances for BMI, showing a *p*-value of < 0.01, suggesting equal variances for two groups. Therefore, homogeneity of the data can be ensured in both cross-validation or BMI groupings. BMI was categorized according to the WHO BMI Classification: BMI ≤ 18.5 kg/m^2^ as underweight, between 18.5 and 24.9 kg/m^2^ as normal, between 25 and 29.9 kg/m^2^ as overweight and ≥30 kg/m^2^ as obesity (40). AIC is a penalized likelihood, balanced between model fit and the number of estimated variables (41). AIC has been applied to evaluate the performance of the series of estimation equations in this study. NHS continuing healthcare checklist is a screening tool that can be used to help researchers or medical personnel understand the needs of subjects or patients for medical care. After completing the checklist, the health status of the subjects can be clearly understood. It can be used to determine whether the volunteer can be accepted in this study. In this study, some subjects who did not meet the acceptance criteria need to be excluded, or those who met the research purpose and health conditions. The recruitment of the subjects belongs to the non-random purposive sampling method.

The cross-validation of this study was to use an independent sample separated from the constructed sample of estimation equations to verify the prediction equation. In theory, cross-validation is performed by independent samples consistent with the applicable conditions of the estimation equation. Therefore, in order to meet this condition, all the subjects were randomly divided into 2/3 and 1/3 as the model establishment group and the validation group for cross-validation (42, 43). In addition, we could also apply the k-fold or leave-one-out method to BIA for cross-validation of body composition estimation equations (44, 45). The Least absolute shrinkage and selection operator (LASSO) developed by Tibshirani in 1996 can increase the preset accuracy and interpretability of statistical models (46). Compared with the stepwise regression analysis method used in this study, each has its own advantages in different application conditions (47). Therefore, in order to improve the performance of the estimation model established in the future, LASSO is one of the statistical suitable methods that can be selected.

This study has a few limitations, first that it included only Asian ethnic groups in Taiwan, which may limit generalization to other populations or ethnic groups; whether the results of this study are applicable to other ethnic groups needs to be further explored. In addition, only adults were selected as subjects, and the hydration status of children and adolescents differs from that of adults. Therefore, the SFA estimation model established in this study is not applicable to subjects under the age of 20. Because of the limitations of funding and manpower, this study cannot conduct research with a large, and multiple sampling method in different medical fields. Therefore, it cannot fully solve the problem of external validity, which is the limitation of this study. To increase the adequacy of cross-validation, similar studies may consider applying LASSO for model selection, or using k-fold or leave-one-out method in future studies.

## Data availability statement

The raw data supporting the conclusions of this article will be made available by the authors, without undue reservation.

## Ethics statement

The studies involving human participants were reviewed and approved by the Human Trials Committee of Taso-Tun Psychiatric Center, Ministry of Health and Welfare, Nan-Tou, Taiwan (IRB 109043). The patients/participants provided their written informed consent to participate in this study.

## Author contributions

C-LL, H-KL, and K-CH interpreted the results, critically reviewed the manuscript, and supervised the study. K-CH designed the study and conducted the research. C-LL, L-PC, and K-CH contributed to subject recruitment and data collection. H-KL, C-LL, A-CH, H-YC, and K-CH performed the laboratory analysis and statistical analysis, interpreted the results, and wrote the manuscript. All authors read and approved the final manuscript.

## Funding

This study was supported by a grant from Research and Development Award Program of the Ministry of Health and Welfare with the award number of PG11001-0566.

## Conflict of interest

Author K-CH was employed by Department of Research and Development, Starbia Meditek Co., Ltd. The remaining authors declare that the research was conducted in the absence of any commercial or financial relationships that could be construed as a potential conflict of interest.

## Publisher's note

All claims expressed in this article are solely those of the authors and do not necessarily represent those of their affiliated organizations, or those of the publisher, the editors and the reviewers. Any product that may be evaluated in this article, or claim that may be made by its manufacturer, is not guaranteed or endorsed by the publisher.
